# Crystal structure of (2*E*)-1-(1-benzo­furan-2-yl)-3-(2-bromo­phen­yl)prop-2-en-1-one monohydrate

**DOI:** 10.1107/S2056989015018897

**Published:** 2015-10-14

**Authors:** S. Satheeshchandra, Nandakumar Shetty

**Affiliations:** a Department of Physics, S. D. M College (Autonomous), Ujire 574 240, India

**Keywords:** crystal structure, benzo­furan, chiral, NLO properties, hydrogen bonding

## Abstract

The title compound, C_17_H_11_BrO_2_·H_2_O, crystallizes as a monohydrate in the chiral ortho­rhom­bic space group *P*2_1_2_1_2_1_, and has non-linear optical (NLO) properties. The mol­ecule has an *E* conformation about the C=C bond and is relatively planar with the benzo­furan and bromo­phenyl rings being inclined to one another by 10.60 (14)°. In the crystal, the water mol­ecule is linked to the organic mol­ecule by O—H⋯O hydrogen bonds, forming an *R* ^2^_2_(7) ring motif while C—H⋯O hydrogen bonds lead to the formation of helices along the *b*-axis direction.

## Related literature   

For background to chalcones and their biological and other properties, see: Choudary *et al.* (1999 ▸); Jayarama *et al.* (2013 ▸); Tomazela *et al.* (2000 ▸); Gu *et al.* (2008 ▸). For the crystal structure of a similar compound, see: Benmekhbi *et al.* (2009 ▸).
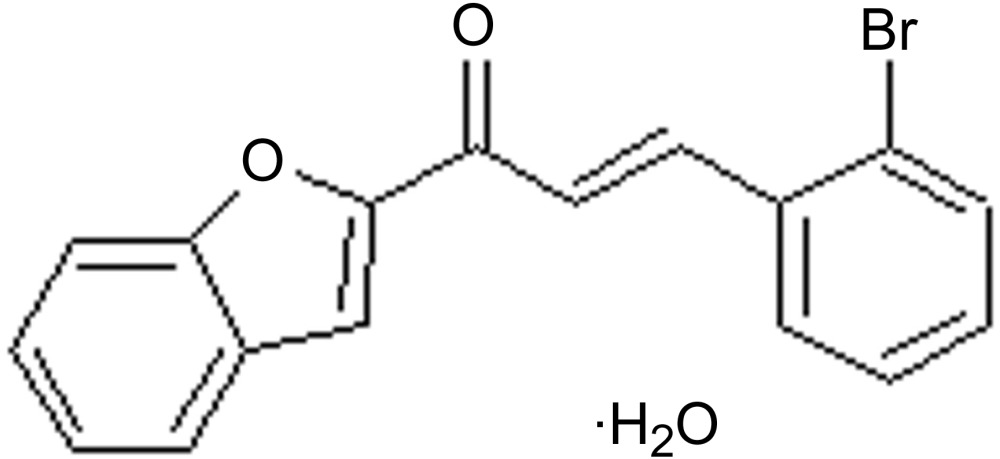


## Experimental   

### Crystal data  

C_17_H_11_BrO_2_·H_2_O
*M* *_r_* = 345.18
Orthorhombic, 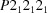

*a* = 4.8614 (4) Å
*b* = 13.8220 (15) Å
*c* = 21.755 (2) Å
*V* = 1461.8 (2) Å^3^
*Z* = 4
Mo *K*α radiation
μ = 2.82 mm^−1^
*T* = 296 K
0.40 × 0.30 × 0.25 mm

### Data collection  

Bruker Kappa APEXII CCD diffractometer
Absorption correction: multi-scan (*SADABS*; Bruker, 2004 ▸) *T*_min_ = 0.399, *T*_max_ = 0.539
3636 measured reflections
3636 independent reflections
2134 reflections with *I* > 2σ(*I*)
*R*_int_ = 0.034

### Refinement  

*R*[*F* ^2^ > 2σ(*F* ^2^)] = 0.042
*wR*(*F* ^2^) = 0.102
*S* = 1.01
3636 reflections
196 parameters
3 restraints
H atoms treated by a mixture of independent and constrained refinement
Δρ_max_ = 0.42 e Å^−3^
Δρ_min_ = −0.29 e Å^−3^
Absolute structure: Flack (1983 ▸), 1497 Friedel pairs
Absolute structure parameter: −0.011 (11)

### 

Data collection: *APEX2* (Bruker, 2004 ▸); cell refinement: *APEX2* and *SAINT* (Bruker, 2004 ▸); data reduction: *SAINT* and *XPREP* (Bruker, 2004 ▸); program(s) used to solve structure: *SIR92* (Altomare *et al.*, 1994 ▸); program(s) used to refine structure: *SHELXL97* (Sheldrick, 2008 ▸); molecular graphics: *Mercury* (Macrae *et al.*, 2008 ▸); software used to prepare material for publication: *SHELXL97*.

## Supplementary Material

Crystal structure: contains datablock(s) I, global. DOI: 10.1107/S2056989015018897/su5214sup1.cif

Structure factors: contains datablock(s) I. DOI: 10.1107/S2056989015018897/su5214Isup3.hkl

Supporting information file. DOI: 10.1107/S2056989015018897/su5214Isup3.cml

**Fig. 1.**
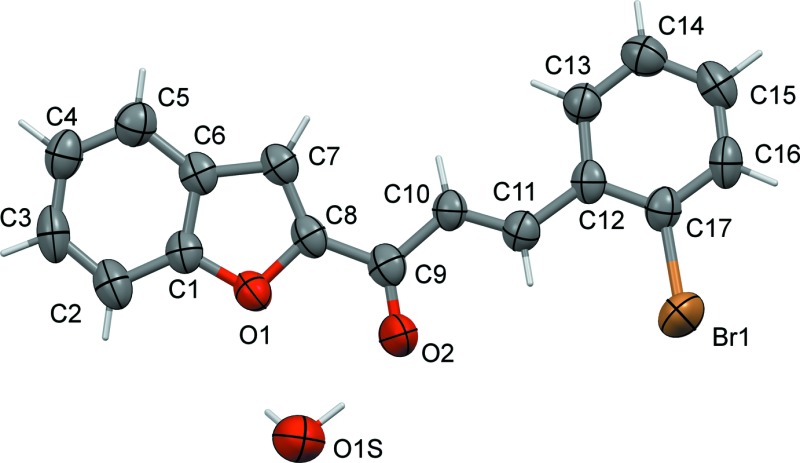
A view of the molecular structure of title compound, with atom labelling. Displacement ellipsoids are drawn at the 50% probability level.

**Fig. 2.**
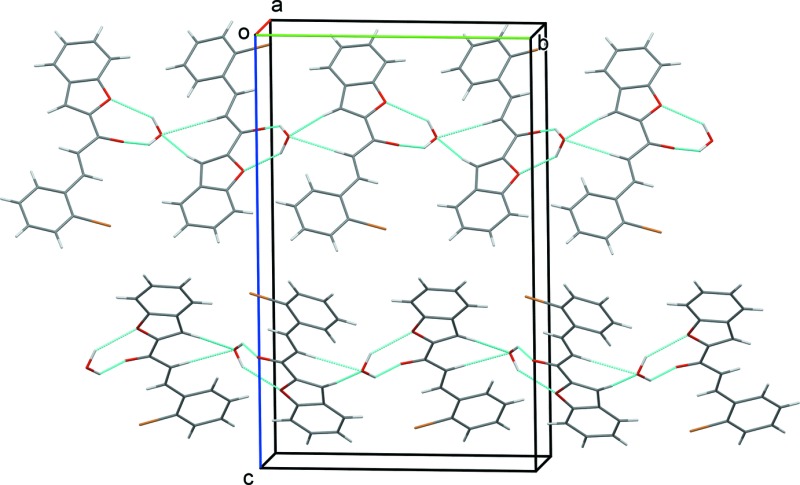
A view along the a-axis of the crystal packing of the title compound. The intermolecular interactions are represented by dashed lines (see Table 1).

. DOI: 10.1107/S2056989015018897/su5214fig1.tif
A view of the mol­ecular structure of title compound, with atom labelling. Displacement ellipsoids are drawn at the 50% probability level.

a . DOI: 10.1107/S2056989015018897/su5214fig2.tif
A view along the *a*-axis of the crystal packing of the title compound. The inter­molecular inter­actions are represented by dashed lines (see Table 1).

CCDC reference: 1430039

Additional supporting information:  crystallographic information; 3D view; checkCIF report

## Figures and Tables

**Table 1:** Hydrogen-bond geometry (, )

*D*H*A*	*D*H	H*A*	*D* *A*	*D*H*A*
O1*S*H1*SA*O1	0.96(2)	2.37(4)	3.181(4)	142(4)
O1*S*H1*SB*O2	0.95(2)	2.11(4)	2.928(4)	143(4)
C7H7O1*S* ^i^	0.93	2.32	3.229(5)	164
C10H10O1*S* ^i^	0.93	2.45	3.375(5)	174

Symmetry code: (i) 
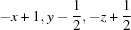
.

## supplementary crystallographic information

### S1. Commentary

Chalcones are among the most abundant and ubiquitous group of natural products (Tomazela, *et al.*, 2000). Some of the chalcone derivatives shows high second-harmonic generation conversion efficiency (Gu *et al.*, 2008; Choudary *et al.*, 1999; Jayarama *et al.*, 2013). The title compound crystallizes in a non-centrosymmteric space group and exhibits inter­esting nonlinear optical properties.

In the title compound, Fig. 1, the benzo­furan and bromo­phenyl groups are linked by a prop-2-en-1-one group. The benzo­furan and bromo­phenyl rings are slightly non-planar with a dihedral angle of 10.60 (14) °. The torsion angle C7–C8–C9–O2 is 175.9 (4)° indicating that the O3 meth­oxy group is coplanar with the attached benzo­furan ring. The C═C double bond shows an *E* conformation.

In the crystal, the water molecule is linked to the organic molecule by O—H···O hydrogen bonds forming an *R*^2^_2_(7) ring motif (Table 1 and Fig. 2). The organic molecule is linked to the water molecule by C—H.·O hydrogen bonds so forming helices along the *b*-axis direction (Table and Fig. 2).

### S2. Synthesis and crystallization

2-Benzo­furanyl methyl ketone (0.01 mol) and 2-bromo­benzaldehyde (0.01 mol) were dissolved in methanol (60 ml). Sodium hydroxide (2 ml, 20%) was then added drop wise to the solution which was then stirred for 1.5 h. The contents of the flask were cooled using ice-cold water, and the resulting powder content was collected by filtration. The compound was dried and re-crystallized twice using acetone as solvent, yielding golden-yellow block-like crystals.

### S3. Refinement

Crystal data, data collection and structure refinement details are summarized in Table 2. The water H atoms were located in a difference Fourier map. They were refined with distance restraints: O—H = 0.88 (2) Å, H···H = 1.43 (2) Å with *U*_iso_(H) = 1.5*U*_eq_(O). The C-bound H atoms were placed in calculated positions and included in the refinement in the riding model approximation: C—H = 0.93 Å with *U*_iso_(H) = 1.2*U*_eq_(C).

### Crystal data

**Table:**

C_17_H_11_BrO_2_·H_2_O	*F*(000) = 696
*M**_r_* = 345.18	*D*_x_ = 1.568 Mg m^−^^3^
Orthorhombic, *P*2_1_2_1_2_1_	Mo *K*α radiation, λ = 0.71073 Å
Hall symbol: P 2ac 2ab	Cell parameters from 2499 reflections
*a* = 4.8614 (4) Å	θ = 4.8–41.6°
*b* = 13.8220 (15) Å	µ = 2.82 mm^−^^1^
*c* = 21.755 (2) Å	*T* = 296 K
*V* = 1461.8 (2) Å^3^	Block, golden-yellow
*Z* = 4	0.40 × 0.30 × 0.25 mm

### Data collection

**Table:**

Bruker Kappa APEXII CCD diffractometer	3636 independent reflections
Radiation source: fine-focus sealed tube	2134 reflections with *I* > 2σ(*I*)
Graphite monochromator	*R*_int_ = 0.034
ω and φ scan	θ_max_ = 28.4°, θ_min_ = 3.0°
Absorption correction: multi-scan (*SADABS*; Bruker, 2004)	*h* = −6→5
*T*_min_ = 0.399, *T*_max_ = 0.539	*k* = −18→18
3636 measured reflections	*l* = −28→29

### Refinement

**Table:**

Refinement on *F*^2^	Secondary atom site location: difference Fourier map
Least-squares matrix: full	Hydrogen site location: inferred from neighbouring sites
*R*[*F*^2^ > 2σ(*F*^2^)] = 0.042	H atoms treated by a mixture of independent and constrained refinement
*wR*(*F*^2^) = 0.102	*w* = 1/[σ^2^(*F*_o_^2^) + (0.0372*P*)^2^ + 0.0197*P*] where *P* = (*F*_o_^2^ + 2*F*_c_^2^)/3
*S* = 1.01	(Δ/σ)_max_ = 0.001
3636 reflections	Δρ_max_ = 0.42 e Å^−^^3^
196 parameters	Δρ_min_ = −0.29 e Å^−^^3^
3 restraints	Absolute structure: Flack (1983), 1497 Friedel pairs
Primary atom site location: structure-invariant direct methods	Absolute structure parameter: −0.011 (11)

### Special details

**Table:**

Geometry. All e.s.d.'s (except the e.s.d. in the dihedral angle between two l.s. planes) are estimated using the full covariance matrix. The cell e.s.d.'s are taken into account individually in the estimation of e.s.d.'s in distances, angles and torsion angles; correlations between e.s.d.'s in cell parameters are only used when they are defined by crystal symmetry. An approximate (isotropic) treatment of cell e.s.d.'s is used for estimating e.s.d.'s involving l.s. planes.
Refinement. Refinement of *F*^2^ against ALL reflections. The weighted *R*-factor *wR* and goodness of fit *S* are based on *F*^2^, conventional *R*-factors *R* are based on *F*, with *F* set to zero for negative *F*^2^. The threshold expression of *F*^2^ > σ(*F*^2^) is used only for calculating *R*-factors(gt) *etc*. and is not relevant to the choice of reflections for refinement. *R*-factors based on *F*^2^ are statistically about twice as large as those based on *F*, and *R*- factors based on ALL data will be even larger.

### Fractional atomic coordinates and isotropic or equivalent isotropic displacement parameters (Å^2^)

**Table:**

	*x*	*y*	*z*	*U*_iso_*/*U*_eq_	
Br1	1.34723 (10)	0.98219 (3)	0.06738 (2)	0.0902 (2)	
O1	0.3289 (4)	0.93267 (17)	0.32535 (10)	0.0513 (5)	
O2	0.6697 (5)	0.99535 (19)	0.23842 (13)	0.0720 (7)	
C1	0.1694 (7)	0.8801 (2)	0.36525 (14)	0.0463 (8)	
C2	−0.0186 (7)	0.9171 (3)	0.40591 (16)	0.0593 (10)	
H2	−0.0523	0.9831	0.4093	0.071*	
C3	−0.1522 (7)	0.8509 (3)	0.44089 (16)	0.0634 (10)	
H3	−0.2812	0.8724	0.4694	0.076*	
C4	−0.1034 (7)	0.7519 (3)	0.43576 (17)	0.0607 (9)	
H4	−0.2020	0.7091	0.4603	0.073*	
C5	0.0860 (7)	0.7169 (3)	0.39547 (16)	0.0587 (10)	
H5	0.1185	0.6507	0.3923	0.070*	
C6	0.2301 (6)	0.7824 (3)	0.35908 (14)	0.0444 (8)	
C7	0.4402 (6)	0.7757 (3)	0.31357 (15)	0.0468 (8)	
H7	0.5256	0.7195	0.2997	0.056*	
C8	0.4907 (6)	0.8671 (3)	0.29448 (15)	0.0463 (9)	
C9	0.6756 (7)	0.9078 (3)	0.24929 (15)	0.0503 (8)	
C10	0.8640 (6)	0.8424 (2)	0.21760 (14)	0.0446 (7)	
H10	0.8712	0.7779	0.2297	0.054*	
C11	1.0223 (6)	0.8713 (3)	0.17288 (16)	0.0480 (8)	
H11	1.0081	0.9361	0.1619	0.058*	
C12	1.2190 (6)	0.8136 (3)	0.13815 (14)	0.0439 (8)	
C13	1.2629 (7)	0.7170 (3)	0.15197 (16)	0.0523 (9)	
H13	1.1617	0.6892	0.1837	0.063*	
C14	1.4478 (7)	0.6610 (3)	0.12115 (19)	0.0615 (10)	
H14	1.4691	0.5961	0.1315	0.074*	
C15	1.6028 (7)	0.7008 (3)	0.07476 (17)	0.0603 (10)	
H15	1.7313	0.6633	0.0539	0.072*	
C16	1.5667 (7)	0.7964 (3)	0.05931 (16)	0.0557 (9)	
H16	1.6695	0.8239	0.0278	0.067*	
C17	1.3787 (7)	0.8507 (3)	0.09068 (14)	0.0493 (8)	
O1S	0.1601 (7)	1.1082 (2)	0.23870 (17)	0.0950 (10)	
H1SA	0.179 (11)	1.078 (4)	0.2780 (12)	0.143*	
H1SB	0.300 (9)	1.070 (3)	0.220 (2)	0.143*	

### Atomic displacement parameters (Å^2^)

**Table:**

	*U*^11^	*U*^22^	*U*^33^	*U*^12^	*U*^13^	*U*^23^
Br1	0.1130 (4)	0.0695 (3)	0.0881 (3)	0.0070 (3)	0.0327 (3)	0.0255 (2)
O1	0.0458 (12)	0.0526 (14)	0.0555 (14)	0.0017 (11)	0.0037 (12)	−0.0104 (11)
O2	0.0594 (14)	0.0592 (19)	0.0974 (19)	0.0071 (14)	0.0271 (13)	0.0068 (15)
C1	0.0391 (17)	0.057 (2)	0.0426 (18)	−0.0028 (18)	−0.0028 (16)	−0.0049 (15)
C2	0.0461 (19)	0.079 (3)	0.053 (2)	0.0023 (19)	0.0023 (17)	−0.016 (2)
C3	0.0426 (18)	0.104 (3)	0.044 (2)	−0.003 (2)	0.0102 (19)	−0.009 (2)
C4	0.056 (2)	0.081 (3)	0.0447 (19)	−0.0068 (19)	0.002 (2)	0.008 (2)
C5	0.058 (2)	0.065 (2)	0.054 (2)	0.0003 (19)	−0.0023 (18)	0.0054 (19)
C6	0.0340 (17)	0.058 (2)	0.0408 (17)	0.0013 (15)	−0.0096 (14)	−0.0056 (16)
C7	0.0383 (17)	0.056 (2)	0.0460 (19)	0.0050 (15)	−0.0053 (15)	−0.0044 (17)
C8	0.0323 (16)	0.060 (2)	0.047 (2)	0.0051 (16)	−0.0044 (14)	−0.0082 (18)
C9	0.0368 (17)	0.060 (2)	0.054 (2)	−0.0001 (18)	−0.0042 (16)	−0.0041 (17)
C10	0.0346 (16)	0.052 (2)	0.0469 (18)	−0.0021 (17)	0.0011 (16)	0.0002 (15)
C11	0.0423 (16)	0.055 (2)	0.047 (2)	0.0008 (16)	−0.0021 (16)	0.0048 (16)
C12	0.0302 (16)	0.060 (2)	0.0419 (18)	−0.0028 (15)	−0.0042 (13)	−0.0007 (16)
C13	0.050 (2)	0.056 (2)	0.051 (2)	0.0004 (17)	0.0043 (15)	0.0046 (18)
C14	0.059 (2)	0.057 (2)	0.068 (3)	0.0079 (19)	−0.003 (2)	−0.001 (2)
C15	0.051 (2)	0.074 (3)	0.055 (2)	0.0128 (19)	0.0024 (18)	−0.014 (2)
C16	0.0437 (18)	0.085 (3)	0.0389 (19)	−0.0034 (18)	0.0069 (15)	0.0009 (19)
C17	0.0434 (18)	0.063 (2)	0.0418 (18)	−0.0013 (18)	−0.0009 (15)	0.0050 (15)
O1S	0.098 (2)	0.071 (2)	0.117 (3)	0.0067 (18)	0.019 (2)	0.0037 (18)

### Geometric parameters (Å, º)

**Table:**

Br1—C17	1.893 (4)	C9—C10	1.460 (5)
O1—C1	1.372 (4)	C10—C11	1.303 (4)
O1—C8	1.375 (4)	C10—H10	0.9300
O2—C9	1.234 (4)	C11—C12	1.456 (4)
C1—C2	1.370 (5)	C11—H11	0.9300
C1—C6	1.390 (5)	C12—C13	1.385 (5)
C2—C3	1.356 (5)	C12—C17	1.390 (4)
C2—H2	0.9300	C13—C14	1.363 (5)
C3—C4	1.393 (5)	C13—H13	0.9300
C3—H3	0.9300	C14—C15	1.374 (5)
C4—C5	1.361 (5)	C14—H14	0.9300
C4—H4	0.9300	C15—C16	1.375 (5)
C5—C6	1.392 (5)	C15—H15	0.9300
C5—H5	0.9300	C16—C17	1.366 (5)
C6—C7	1.426 (4)	C16—H16	0.9300
C7—C8	1.353 (5)	O1S—H1SA	0.958 (19)
C7—H7	0.9300	O1S—H1SB	0.954 (19)
C8—C9	1.446 (5)		
			
C1—O1—C8	106.5 (3)	C8—C9—C10	118.1 (3)
C2—C1—O1	126.0 (3)	C11—C10—C9	122.2 (3)
C2—C1—C6	124.5 (3)	C11—C10—H10	118.9
O1—C1—C6	109.5 (3)	C9—C10—H10	118.9
C3—C2—C1	115.5 (4)	C10—C11—C12	127.4 (4)
C3—C2—H2	122.2	C10—C11—H11	116.3
C1—C2—H2	122.2	C12—C11—H11	116.3
C2—C3—C4	122.4 (3)	C13—C12—C17	115.6 (3)
C2—C3—H3	118.8	C13—C12—C11	121.1 (3)
C4—C3—H3	118.8	C17—C12—C11	123.4 (3)
C5—C4—C3	121.1 (3)	C14—C13—C12	122.8 (3)
C5—C4—H4	119.4	C14—C13—H13	118.6
C3—C4—H4	119.4	C12—C13—H13	118.6
C4—C5—C6	118.4 (4)	C13—C14—C15	119.7 (4)
C4—C5—H5	120.8	C13—C14—H14	120.2
C6—C5—H5	120.8	C15—C14—H14	120.2
C1—C6—C5	118.1 (3)	C14—C15—C16	119.7 (3)
C1—C6—C7	106.4 (3)	C14—C15—H15	120.2
C5—C6—C7	135.5 (4)	C16—C15—H15	120.2
C8—C7—C6	106.4 (3)	C17—C16—C15	119.4 (3)
C8—C7—H7	126.8	C17—C16—H16	120.3
C6—C7—H7	126.8	C15—C16—H16	120.3
C7—C8—O1	111.2 (3)	C16—C17—C12	122.8 (3)
C7—C8—C9	133.2 (3)	C16—C17—Br1	116.6 (3)
O1—C8—C9	115.6 (3)	C12—C17—Br1	120.6 (3)
O2—C9—C8	119.8 (3)	H1SA—O1S—H1SB	94 (2)
O2—C9—C10	122.1 (3)		
			
C8—O1—C1—C2	178.7 (3)	O1—C8—C9—O2	−3.8 (5)
C8—O1—C1—C6	−0.4 (3)	C7—C8—C9—C10	−3.6 (6)
O1—C1—C2—C3	−179.9 (3)	O1—C8—C9—C10	176.5 (3)
C6—C1—C2—C3	−1.0 (5)	O2—C9—C10—C11	−5.2 (5)
C1—C2—C3—C4	−0.3 (5)	C8—C9—C10—C11	174.5 (3)
C2—C3—C4—C5	1.0 (6)	C9—C10—C11—C12	179.5 (3)
C3—C4—C5—C6	−0.3 (5)	C10—C11—C12—C13	−2.0 (5)
C2—C1—C6—C5	1.6 (5)	C10—C11—C12—C17	179.6 (3)
O1—C1—C6—C5	−179.3 (3)	C17—C12—C13—C14	−0.5 (5)
C2—C1—C6—C7	−178.3 (3)	C11—C12—C13—C14	−179.0 (3)
O1—C1—C6—C7	0.8 (3)	C12—C13—C14—C15	1.0 (5)
C4—C5—C6—C1	−0.9 (5)	C13—C14—C15—C16	−1.0 (5)
C4—C5—C6—C7	179.0 (3)	C14—C15—C16—C17	0.5 (5)
C1—C6—C7—C8	−0.9 (3)	C15—C16—C17—C12	−0.1 (5)
C5—C6—C7—C8	179.1 (3)	C15—C16—C17—Br1	178.9 (3)
C6—C7—C8—O1	0.8 (4)	C13—C12—C17—C16	0.1 (5)
C6—C7—C8—C9	−179.1 (3)	C11—C12—C17—C16	178.6 (3)
C1—O1—C8—C7	−0.3 (4)	C13—C12—C17—Br1	−178.9 (2)
C1—O1—C8—C9	179.6 (3)	C11—C12—C17—Br1	−0.4 (4)
C7—C8—C9—O2	176.0 (3)		

### Hydrogen-bond geometry (Å, º)

**Table:**

*D*—H···*A*	*D*—H	H···*A*	*D*···*A*	*D*—H···*A*
O1*S*—H1*SA*···O1	0.96 (2)	2.37 (4)	3.181 (4)	142 (4)
O1*S*—H1*SB*···O2	0.95 (2)	2.11 (4)	2.928 (4)	143 (4)
C7—H7···O1*S*^i^	0.93	2.32	3.229 (5)	164
C10—H10···O1*S*^i^	0.93	2.45	3.375 (5)	174

Symmetry code: (i) −*x*+1, *y*−1/2, −*z*+1/2.
