# Supplementary figures and images for: Crystal structure of (2E)-1-(1-benzo­furan-2-yl)-3-(2-bromo­phen­yl)prop-2-en-1-one monohydrate
Source: Acta Crystallogr E Crystallogr Commun. 2015 Oct 14;71(Pt 11):o840–1. doi: 10.1107/S2056989015018897 (PMC4645062; doi:10.1107/S2056989015018897)

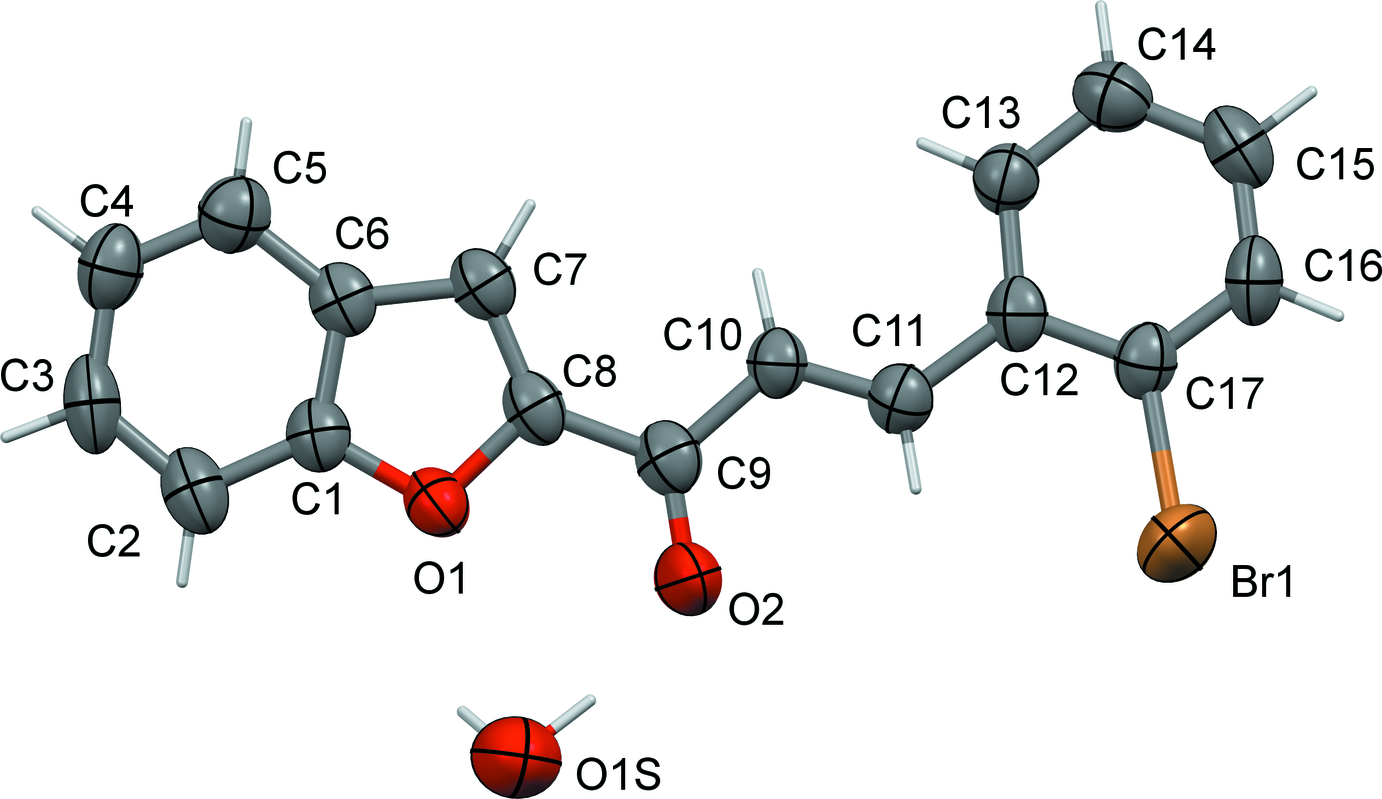

Supplement: Supplementary file 4 [file e-71-0o840-fig1.tif]

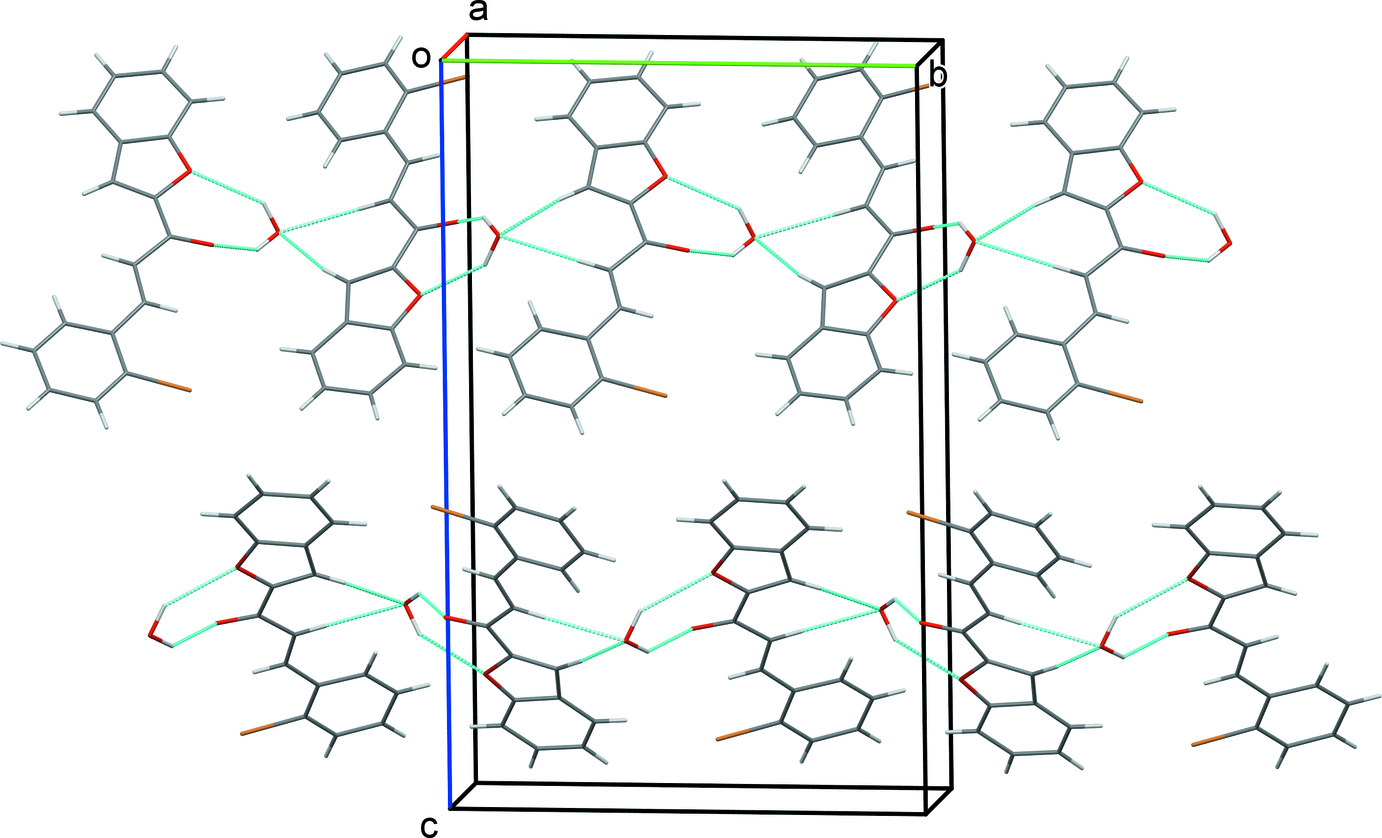

Supplement: Supplementary file 5 [file e-71-0o840-fig2.tif]
